# Evaluation of ultrasound-guided distal catheter placement in pediatric ventriculoatrial shunts for patients with hydrocephalus: Effectiveness and consequences

**DOI:** 10.1177/11297298251317568

**Published:** 2025-02-12

**Authors:** Giovanni Rollo, Francesca Maria Silvestri, Giorgio Persano, Angela Mastronuzzi, Andrea Carai, Carlo Efisio Marras, Antonella Cacchione, Silvia Madafferi, Cristina Martucci, Simone Reali, Chiara Grimaldi, Gian Luigi Natali, Daniella Araiza Kelly, Alessandro Crocoli

**Affiliations:** 1 Surgical Oncology Unit, Bambino Gesù Pediatric Hospital IRCCS, Rome, Italy; 2 Pediatric Haematology and Oncology, and Cell and Gene Therapy Unit, Bambino Gesù Children’s Hospital, IRCCS, Rome, Italy; 3 Neurosurgery Unit, Bambino Gesù Children’s Hospital, IRCCS, Rome, Italy; 4 Anesthesia and Critical Care Unit, Bambino Gesù Children’s Hospital, IRCCS, Rome, Italy; 5 Diagnostic and Interventional Radiology Unit, Bambino Gesù Children’s Hospital, IRCCS, Rome, Italy

**Keywords:** Hydrocephalus, ventriculoatrial shunt, ultrasound-guided, pediatric neurosurgery, minimally invasive surgery

## Abstract

**Background and aims:**

Ventriculoatrial (VA) shunts are frequently used for hydrocephalus (HS) management when peritoneal catheter placement is inappropriate. Historically, open surgical cut-down (OSC) on the internal jugular vein has been the standard method for distal catheter insertion. In contrast, percutaneous Seldinger-type ultrasound-guided (USG) venipuncture offers advantages such as reduced operating times and lower postoperative pain. However, its use in pediatric patients is limited.

**Methods:**

This study reviewed patients diagnosed with HS who underwent VA shunt procedures (OSC vs USG) at Bambino Gesù Children’s Hospital from January 1, 2014, to February 29, 2024. The analysis focused on surgical times for VA shunt placements and associated neurosurgical operations, as well as catheter replacement rates.

**Results:**

Thirteen patients (6 males, 7 females; median age 12 years, range 0.5–14.2) were enrolled, with a total of 23 procedures performed. The mean surgical time for distal VA placement using the USG technique was significantly shorter than for the OSC method (13.36 min vs 30.22 min, *p* = 0.00001). Conversely, neurosurgical operations performed using OSC had a 15-min reduction in average operative time compared to USG, though this difference was not statistically significant. Catheter replacement was required in 35.7% of the USG group compared to 55.5% in the OSC group (*p* = ns).

**Conclusions:**

USG VA shunt placement demonstrates reduced operating times and lower perioperative complication rates compared to OSC. Our findings indicate that percutaneous VA shunts are technically simpler and do not necessitate specialized pediatric vascular surgery skills, enhancing their applicability in pediatric hydrocephalus management.

## Introduction

Hydrocephalus (HS) is a complex neurological condition characterized by the accumulation of cerebrospinal fluid (CSF) within the ventricles of the brain.^1^ This buildup leads to an increase in intracranial pressure, which can cause neurological impairment. Proper management of hydrocephalus is essential to prevent long-term complications, such as cognitive impairment, developmental delays, and motor deficits. Ventriculoatrial (VA) shunting has emerged as a preferred management option when traditional pathways, such as ventricular-peritoneal (VP) shunts, are not feasible due to anatomical or pathological factors.^2^

The open surgical cut-down (OSC) approach has historically been considered the traditional method for inserting distal catheters into the internal jugular vein to manage HS.^2,3^ This technique, although effective, has several disadvantages, including longer operative time, increased postoperative discomfort, and a higher probability of complications related to more invasive surgical techniques. Additionally, the cut-down technique often necessitates the sacrifice of the vein, which poses a significant limitation in pediatric patients requiring future shunt revisions.^4^ The availability of this procedure also may be limited in certain hospital environments due to the necessity for specialized surgical competence.

In recent years, advancements in minimally invasive techniques have led to the introduction of ultrasound-guided (USG) venipuncture and the Seldinger technique for the insertion of VA shunts.^5,6^ Using real-time ultrasound imaging is a novel strategy designed to improve the accuracy of catheter placement while simultaneously reducing the amount of tissue damage.

The anticipated advantages of USG, such as decreased surgery times, lower incidences of perioperative complications, and diminished postoperative pain, are projected to enhance overall patient recovery trajectories and satisfaction.

The objective of the present study was to conduct a comprehensive comparison of the clinical outcomes associated with VA shunt placements conducted using the USG technique and those implanted via the OSC approach in a pediatric population. Based on our experience, we believe that using USG can result in significantly shorter surgical timeframes and lower complication rates, ultimately improving the overall surgical experience for patients and healthcare providers.

## Materials and methods

This retrospective cohort study was conducted at our tertiary referral center (Bambino Gesù Children’s Hospital, IRCCS, Rome, Italy). Included in the study were pediatric patients diagnosed with hydrocephalus who underwent ventriculoatrial (VA) shunt placement between January 1, 2014, and February 29, 2024. Ethical approval was granted by the hospital’s institutional review board, ensuring compliance with ethical standards and safeguarding patient confidentiality.

### Study population and inclusion criteria

Inclusion criteria were pediatric patients diagnosed with HS necessitating VA shunt placement, regardless of underlying etiology, which included congenital anomalies, traumatic injuries, or secondary complications from infections or neoplasms. Exclusion criteria were: patients with incomplete medical records, patients undergoing concurrent unrelated surgical procedures, and emergency cases where the shunt placement method could not be predetermined.

### Procedures

The cranial part of the procedure does not differ between the two patient cohorts studied. Under general anesthesia, the patient’s head is positioned on a soft pad and rotated toward the side opposite to the shunt. Real time frameless navigation is used in all cases (StealthStation^®^ S8, Medtronic, Fort Worth, TX, USA) to position a frontal horn ventricular catheter through a burr hole on Kocher’s point. The catheter is then connected to a subcutaneous reservoir (Holter^®^ Rickham^®^ reservoir, Integra Lifesciences, Mansfield, MA, USA) to ensure stability during subsequent surgical maneuvers. The subcutaneous reservoir is attached to a valve system and to a distal atrial catheter (Codman^®^ Holter^®^ Atrial Catheter “Type E,” Integra Lifesciences, Mansfield, MA, USA) that has been tunneled from a designated neck site to subsequently approach the jugular vein. Custom length of the atrial catheter is determined on the sterile field before the beginning of the procedure by anatomical landmarks. The system is therefore positioned in the subcutaneous plane with the distal catheter exteriorized at the neck.

#### Ultrasound guided procedure

**Figure 1.**
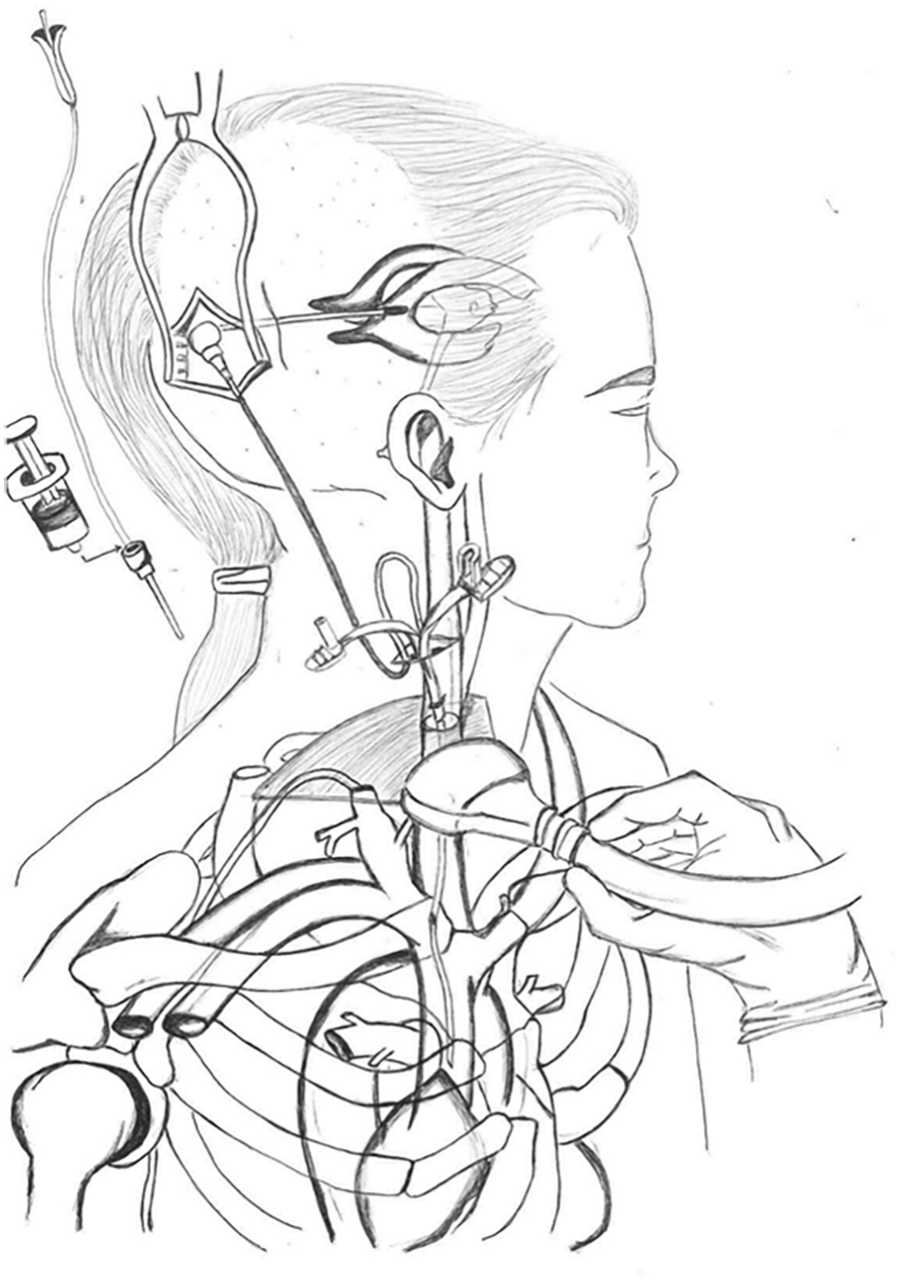
Schematic representation of ultrasound-guided ventriculoatrial shunt placement. This illustration is a modified version of the original figure published in Metellus et al.^7^ Modifications were made to enhance clarity and highlight specific aspects relevant to the current study. Reproduction of the image in this work has been authorized in accordance with the journal’s copyright policies. Authorization documentation is available upon request.

The combined procedure for ultrasound-guided venipuncture of the internal jugular vein or brachiocephalic vein during VA shunt placement involves a systematic approach that enhances the accuracy and safety of catheterization procedures. Following appropriate patient positioning and sterile preparation, high-frequency ultrasound is used to identify the vascular anatomy, including the internal jugular vein (IJV) and its relationship with surrounding structures such as the carotid artery and the brachiocephalic vein. Once anatomical landmarks are identified, a suitable access site is selected, typically where the internal jugular vein (IJV) is most superficial and accessible. Therefore, the ultrasound probe (Sonosite SII—VA with L25x linear transducer, Fujifilm Sonosite, Bothell, WA, USA) is used to confirm vein patency and provide real-time imaging throughout the puncture process. Using a standard Seldinger technique, a 5 cm 21 G needle is inserted under ultrasound guidance into the target vein, ensuring that the needle trajectory is optimized for successful access without compromising adjacent structures. Upon venous puncture confirmation through blood return, a 0.018 inches nitinol guidewire is introduced into the vessel, followed by 4.5 Fr peel-away introducer sheath of the access site to accommodate the placement of the venous catheter. The distal end of the catheter is then advanced into the venous system toward the right atrium (Figure 1). Continuous ultrasound visualization aids in confirming catheter placement, allowing for the avoidance of complications such as arterial puncture or hematoma formation. Appropriate tip location of the catheter was determined using either fluoroscopic guidance, ECG monitoring or echocardiography. Fluoroscopy, however, could be extremely challenging due to the small size of the catheter’s distal tip, which made visualization difficult. As a result, the procedure often necessitated the injection of diluted contrast media to enhance the visibility of the catheter during imaging. Intracavitary ECG has been utilized as an alternative method for tip location, harnessing changes in the P wave as the intracavitary electrode advances through the vein toward the atrium. This approach facilitates precise assessment of the catheter’s position while providing valuable information about the electrical activity near the cavoatrial junction. The catheter is pre-filled with a 0.9% saline solution, functioning as an exploratory electrode at the distal tip. As the catheter approaches the right atrium, the P wave, which signifies atrial depolarization and is the initial wave of the cardiac cycle, progressively increases in amplitude. At the cavo-atrial junction, the P wave reaches its peak height, transitioning to a biphasic form within the right atrium, and ultimately becomes entirely negative once it passes beyond this point.

Both techniques enhance the accuracy and safety of the catheter placement procedure. Following successful placement, the catheter is secured, and appropriate connections are made to the proximal end of the VA shunt.

#### Open surgical cutdown procedure

With this approach, the patient is placed in a supine posture and the neck sterilized and draped under general anesthesia. Transversal incision is made along the medial border of the sternocleidomastoid muscle to expose the IJV. After careful dissection and identification of the IJV, a venotomy is performed to introduce the catheter. The VA shunt catheter is advanced through the IJV into the right atrium, establishing a pathway for CSF drainage from the ventricular system to the systemic circulation. The ventricular catheter is subsequently tunneled to the cranial vault and connected to the lateral ventricular drainage system.

### Data collection procedures

Data from medical records of patients were reviewed, including demographics, age at diagnosis, prenatal sign, genetics, outcomes as well as clinical, medical, and surgical management. Key variables analyzed included the surgical technique employed (USG or OSC), surgical duration for distal VA shunt placement and associated neurosurgical procedures, and rates of catheter replacements postoperatively. Surgical time was measured from the initiation of venipuncture until the successful placement of the catheter. Postoperative outcomes were monitored for complications such as infection, thrombosis, and other vascular-related issues.

### Statistical analysis

Statistical analyses were performed utilizing appropriate software (GraphPad PRISM, GraphPad Software, Boston, MA, USA) to facilitate comparisons of means for surgical times and catheter replacement rates between the two study groups. Continuous variables were assessed using *t*-tests to ascertain statistical significance, while categorical variables were analyzed using chi-squared tests. A *p*-value of <0.05 was deemed statistically significant, allowing for meaningful comparisons between the two surgical techniques. The analyses aimed to elucidate the relative efficacy and safety of the USG technique in comparison to the traditional OSC method.

## Results

A total of 13 pediatric patients were enrolled in the study, 6 males and 7 females, with a median age of 12 years (range: 0.5–14.2 years). The sample reflected a heterogeneous population with various etiologies of hydrocephalus, including congenital malformations, post-infectious sequelae, and traumatic brain injuries (Table 1). In total, 23 VA shunt procedures were performed: 10 via the OSC method and 13 with the USG technique.

**Table 1.** Population of the study.

Total patients	13
Males	6
Females	7
Median age	12 years
Age range	0.5–14.2 years
Etiologies of hydrocephalus	- Congenital malformations
	- Post-infectious sequelae
	- Traumatic brain injuries
Total VA shunt procedures	23
Procedures via OSC method	10
Procedures via USG technique	13

### Surgical time analysis

Mean duration for distal VA placement employing the USG approach was significantly shorter, than that for the OSC method, averaging 13.36 min compared to 30.22 min (*p* < 0.00001). This substantial disparity underscores the potential of the USG technique to enhance surgical efficiency and streamline clinical workflows. Notably, the evaluation of overall operative time for neurosurgical interventions associated with VA shunt placements indicated a reduction of approximately 15 min for the OSC technique; however, this difference was not statistically significant.

### Catheter replacement rates

Overall, 35.7% of patients in the USG cohort required at least one catheter replacements, versus 55.5% in the OSC group. Although this difference did not reach statistical significance (*p* = ns), it suggests the need for more detailed study regarding the long-term efficacy and durability of shunt placements between the two methodologies.

### Perioperative complications

A comprehensive analysis of perioperative complications revealed a lower incidence of adverse events, including infection, catheter misplacement, and vascular complications in the USG group. These findings suggest a potential advantage of the minimally invasive approach; however, further statistical investigation is required to validate these observations.

Results of analysis are summarized in Table 2.

**Table 2.** Results.

Results	USG technique	OSC method	Statistical significance
Mean duration for distal VA placement	13.36 min	30.22 min	*p* < 0.00001
Reduction in overall operative time	Not specified	Approximately 15 min reduction	*p* = ns
Catheter replacements required	35.7%	55.5%	*p* = ns

## Discussion

This study demonstrates the substantial advantages of utilizing USG techniques for VA shunt insertion in pediatric patients diagnosed with HS.^8^ The notable reduction in surgical duration associated with the USG method in our study not only enhances operational efficiency but also potentially reduces anesthetic exposure. This is particularly crucial in young patients, as prolonged exposure to anesthesia can lead to adverse outcomes, including cognitive and developmental delays.^9^ By minimizing the time under anesthesia, we may reduce these risks, contributing to better overall patient safety and postoperative recovery.

Moreover, although the lower catheter replacement rates observed in the USG cohort did not reach statistical significance, they suggest a potential improvement in shunt longevity and performance. This aspect is vital, considering that the management of hydrocephalus in pediatric patients often necessitates multiple interventions over the course of their lives. The reduction in shunt failures not only leads to fewer surgeries and lower rates of associated risks but also enhances the quality of life for these children, emphasizing the need for effective long-term management strategies.^10^

In VA shunt placement, optimizing catheter tip positioning is essential for effective cerebrospinal fluid drainage and reducing the risk of complications. Fluoroscopy, often used for this purpose, can be inaccurate as it relies on radiological landmarks. The application of cardiac ultrasound provides a non-ionizing, real-time approach for accurately visualizing tip placement within the right atrium, delivering enhanced anatomical detail compared to traditional chest X-ray imaging.^11–13^ Echocardiography, especially using the subcostal view, typically offers a clear identification of the catheter tip, often without the need for a bubble test. In cases of doubt, the bubble test can further confirm catheter positioning using echogenic contrast, improving procedural accuracy and patient safety.^14^ Given that safer and simpler methods should be prioritized in shunt placement, echocardiography (with or without bubble test) or intracavitary ECG should be preferred over fluoroscopy, as they avoid radiation exposure while providing more accurate visualization.

We believe that the intrinsic simplicity and minimally invasive nature of the percutaneous USG method could lead to its broader adoption among surgical practitioners. This shift may alleviate the dependency on specialized pediatric vascular surgeons, thereby improving access to care, especially in resource-constrained settings where surgical expertise is limited.^3,6,7,15–17^ Furthermore, the substantial surgical time difference (approximately 15 min less for the OSC technique in neurosurgical VA shunt placements) highlights the technique’s ability to streamline clinical workflows, even if this difference is not statistically significant. It is important to note that the USG technique requires specific training, which may contribute to improved efficiency through optimized preparation and instrument placement, as well as precise tip location facilitated by the guidance provided by ultrasound.

## Limitations and future directions

Despite the promising findings of this study, it is critical to acknowledge its inherent limitations. The retrospective design raises concerns regarding potential selection bias, as the choice of surgical strategy may have been influenced by specific patient characteristics or institutional protocols. Additionally, the limited sample size restricts the external validity of our results, necessitating cautious interpretation. A more robust understanding of the long-term outcomes associated with each technique is hindered by the relatively short follow-up period.

Future research should prioritize larger sample sizes and the implementation of randomized controlled trials to substantiate these findings and refine clinical guidelines for VA shunt implantation in pediatric populations. Furthermore, multicenter trials could yield a more comprehensive dataset, providing insights into the efficacy and adaptability of the USG approach across diverse healthcare settings. This expansion of research will not only enhance our understanding of the method’s applicability but also facilitate the development of standardized protocols that can improve patient outcomes on a broader scale.

## Conclusion

In summary, based on our study, the ultrasound-guided technique for VA shunt placement appears to present significant advantages over the traditional open surgical cut-down method in pediatric patients diagnosed with HS. The demonstrable reduction in surgical time, coupled with potential decreases in perioperative complications, advocates for the integration of minimally invasive approaches within pediatric neurosurgery. As the field continues to evolve, ongoing research remains essential to further delineate the long-term effectiveness and safety of these methodologies, ultimately enhancing patient care and surgical outcomes. The findings underscore the importance of embracing innovative surgical techniques that prioritize patient safety, minimize invasiveness, and bolster operational efficiency, thereby optimizing the management of pediatric hydrocephalus and ensuring the best possible outcomes for affected children.

## References

[1] Kahle K Kulkarni A Limbrick D , et al. Hydrocephalus in children. Lancet 2016; 387(10020): 788–799. 26256071 10.1016/S0140-6736(15)60694-8

[2] Hanak B Bonow R Harris C , et al. Cerebrospinal fluid shunting complications in children. Pediatr Neurosurg 2017; 52(6): 381–400. 28249297 10.1159/000452840 PMC5915307

[3] Gmeiner M Wagner H van Ouwerkerk W , et al. Long-term outcomes in ventriculoatrial shunt surgery in patients with pediatric hydrocephalus: retrospective single-center study. World Neurosurg 2020; 138: e112–e118. 10.1016/j.wneu.2020.02.035 32061956

[4] Wongsirisuwan M. The long-term patency of the internal jugular vein and the common facial vein as entrance sites for venous access in ventriculoatrial shunts: a comparative analysis from a single-center study. World Neurosurg 2024; 182: e652–e656. 10.1016/j.wneu.2023.12.011 38065357

[5] Ellegaard L Mogensen S Juhler M. Ultrasound-guided percutaneous placement of ventriculoatrial shunts. Childs Nerv Syst 2007; 23(8): 857–862. 17375310 10.1007/s00381-007-0304-y

[6] Clark D Chakraborty A Roebuck D , et al. Ultrasound guided placement of the distal catheter in paediatric ventriculoatrial shunts-an appraisal of efficacy and complications. Childs Nerv Syst 2016; 32(7): 1219–1225. 27207611 10.1007/s00381-016-3120-4 PMC4947480

[7] Metellus P Hsu W Kharkar S , et al. Accuracy of percutaneous placement of a ventriculoatrial shunt under ultrasonography guidance: a retrospective study at a single institution. J Neurosurg 2009; 110(5): 867–870. 19099376 10.3171/2008.10.17674

[8] Kik C Spoor J. Pragmatism in pediatric neurosurgery: more than a pipe dream? A systematic literature review and analysis. World Neurosurg 2022; 161: 418–423. 35505562 10.1016/j.wneu.2021.09.124

[9] Rymarczuk G Keating R Coughlin D , et al. A comparison of ventriculoperitoneal and ventriculoatrial shunts in a population of 544 consecutive pediatric patients. Neurosurgery 2020; 87(1): 80–85. 31586189 10.1093/neuros/nyz387

[10] Panagopoulos D Stranjalis G Gavra M , et al. Current trends in the treatment of pediatric hydrocephalus: a narrative review centered on the indications, safety, efficacy, and long-term outcomes of available treatment modalities. Children (Basel) 2024; 11(11): 1334. 10.3390/children11111334 PMC11593021 39594909

[11] Isaacs AM Krahn D Walker AM , et al. Transesophageal echocardiography-guided ventriculoatrial shunt insertion. Oper Neurosurg (Hagerstown) 2020; 19(1): 25–31. 10.1093/ons/opz353 31811299

[bibr12-11297298251317568] Chui J MacDougall K Ng W. Real-time 3D transesophageal echocardiography for the placement of ventriculoatrial shunt: a case series and technical note. J Neurosurg Anesthesiol. Epub ahead of print 17 January 2024. DOI: 10.1097/ANA.0000000000000952. 38237577

[13] Rossetti F Pittiruti M Lamperti M , et al. The intracavitary ECG method for positioning the tip of central venous access devices in pediatric patients: results of an Italian multicenter study. J Vasc Access 2015; 16(2): 137–143. 25198817 10.5301/jva.5000281

[14] Asada D Morishita Y Kawai Y , et al. Efficacy of bubble contrast echocardiography in detecting pulmonary arteriovenous fistulas in children with univentricular heart after total cavopulmonary connection. Cardiol Young 2020; 30(2): 227–230. 31916529 10.1017/S104795111900324X

[15] Bakhaidar M Wilcox J Sinclair D , et al. Ventriculoatrial shunts: review of technical aspects and complications. World Neurosurg 2022; 158: 158–164. 34775091 10.1016/j.wneu.2021.11.025

[bibr16-11297298251317568] Bidkar P Kannabiran N Chatterjee P. Clinical applications of ultrasound in neurosurgery and neurocritical care: a narrative review. Med J Armed Forces India 2024; 80(1): 16–28. 38239602 10.1016/j.mjafi.2023.06.007 PMC10793236

[17] Ravindra V Riva-Cambrin J Jensen H , et al. Comparing ventriculoatrial and ventriculopleural shunts in pediatric hydrocephalus: a Hydrocephalus Clinical Research Network study. J Neurosurg Pediatr 2024; 34(4): 305–314. 38968629 10.3171/2024.5.PEDS2469 PMC11244699

